# Therapeutic Effects of Stimulating the Melanocortin Pathway in Regulating Ocular Inflammation and Cell Death

**DOI:** 10.3390/biom14020169

**Published:** 2024-01-31

**Authors:** Shudan Wang, Francesca Kahale, Amirreza Naderi, Pier Luigi Surico, Jia Yin, Thomas Dohlman, Yihe Chen, Reza Dana

**Affiliations:** 1 Schepens Eye Research Institute of Massachusetts Eye and Ear, Harvard Medical School, Boston, MA 02114, USA; dr.wangshudan@gmail.com (S.W.); francesca_kahale@meei.harvard.edu (F.K.); amir.naderi@yale.edu (A.N.); psurico@meei.harvard.edu (P.L.S.); jia_yin@meei.harvard.edu (J.Y.); thomas_dohlman@meei.harvard.edu (T.D.); 2 Eye Hospital, The First Affiliated Hospital of Harbin Medical University, Harbin 150001, China

**Keywords:** α-MSH, cyto-protection, immunoregulation, melanocortin receptors, ocular immunity, ocular pathologies

## Abstract

Alpha-melanocyte-stimulating hormone (α-MSH) and its binding receptors (the melanocortin receptors) play important roles in maintaining ocular tissue integrity and immune homeostasis. Particularly extensive studies have demonstrated the biological functions of α-MSH in both immunoregulation and cyto-protection. This review summarizes the current knowledge of both the physiological and pathological roles of α-MSH and its receptors in the eye. We focus on recent developments in the biology of α-MSH and the relevant clinical implications in treating ocular diseases.

## 1. Introduction

Alpha-melanocyte-stimulating hormone (α-MSH) is an evolutionarily conserved 13-amino acid peptide proteolytically cleaved from the precursor pro-opiomelanocortin (POMC). POMC also encodes β-MSH and γ-MSH and the three forms bind to four melanocortin receptors (MC1R, MC3R, MC4R, and MC5R) with different affinities [1]. Melanocortins were initially identified in pituitary cells, and were found to be also synthesized in monocytes, astrocytes, gastrointestinal cells, and keratinocytes, exerting various physiological roles due to the abundance of MCRs in different tissues. α-MSH is well-known for mediating pigmentation; however, accumulating evidence has highlighted its pleotropic properties. Recent preclinical investigations in models mimicking a wide array of inflammatory conditions, including sepsis, acute respiratory distress syndrome, rheumatoid arthritis, inflammatory bowel disease, and encephalitis, demonstrate that α-MSH possesses potent anti-inflammatory and tissue regenerative capacities, providing potential for novel treatment approaches [2,3,4,5,6]. While systemic anti-inflammatory treatment has shown promise, the ocular surface poses unique challenges for the mitigation of inflammation. As an immune-privileged and self-contained surface, the eye requires specialized consideration of epithelial barriers, neural regulation, and tissue-resident immunomodulatory cells in developing anti-inflammatory therapies. Unresolved inflammation stemming from surgery, trauma, infection, or autoimmunity can quickly result in blinding corneal opacification or vascularization. Given the constitutive availability of α-MSH in the eye, and the wide expression of MCRs in different ocular structures, this review summarizes the current data pertaining to the role of α-MSH in mitigating inflammation and the promotion of ocular surface cell survival [7,8,9].

## 2. α-MSH Bioavailability in the Eye

Constitutive expression of α-MSH has been demonstrated in the aqueous and vitreous humors of mammalian eyes [10]. Within ocular tissues, α-MSH production has been attributed to several cell types including retinal pigment epithelium (RPE), ciliary body epithelium, iris pigment epithelium, hyalocytes, and macrophages [11]. These cells contain enzymes capable of cleaving POMC into biologically active melanocortins such as α-MSH. Through signaling pathways involving T cells and macrophages, melanocortins contribute to the regulation of normal anti-inflammatory and immunosuppressive effects in the eye. This includes the modulation of both cellular and humoral immunity. Overall, constitutive α-MSH expression coupled with local production in ocular tissues highlights its immunoregulatory role in maintaining immune privilege and preventing inappropriate inflammation in the eye [12,13,14]. The unique immunoregulatory ocular microenvironment renders the eye an immune-privileged site, characterized by an absence of a mounted immune response to reject an allograft, even in a primed host [15]. Ocular immune privilege is achieved through (1) the active suppression of effector T cells, neutrophils, and macrophages and (2) the alteration of antigens by antigen-presenting cells (APCs) to decrease inflammation and promote regulatory T cell (Treg) activation [16,17,18,19]. The soluble immunomodulatory factors within the ocular microenvironment are found mainly in the aqueous humor, and these factors suppress the activation of inflammatory immunity, further promoting immune privilege, among which are α-MSH, the first described immunomodulating neuropeptide in the eye; vasoactive intestinal peptide (VIP); calcitonin gene-related peptide (CGRP); substance P (SP); and neuropeptide Y (NPY) [20,21,22,23,24,25,26,27,28]. In the healthy eye, there is constitutive expression of α-MSH in the aqueous humor is at a concentration of approximately 10^−11^ M [28]. Absence of α-MSH in depleted aqueous humor results in the loss of its ability to regulate T cell activity. α-MSH serves as the mediator through which the aqueous humor directs the antigen-specific T cell response, shifting it from proinflammatory to regulatory [29,30]. Although α-MSH does not prevent T cell proliferation, it inhibits activated T cells from producing IFN-γ. In this process, transforming growth factor beta (TGF-β), also found in the aqueous humor, helps to enhance the activity of α-MSH-inducing regulatory activity in T cells [30]. It has been shown that regulatory T cells can be generated in vitro by treating antigen-stimulated CD4^+^ T cells with a supplementation of α-MSH at the physiological concentration of 10^−11^ M. The α-MSH-induced Tregs express CD25 and produce TGF-β, but not IL-4 [29,31,32]. In a recent study, α-MSH was also found in tears, and its concentration increased in perennial allergic conjunctivitis (PAC) patients compared with healthy participants [33].

There are five known α-MSH receptors, MC1R to MC5R. All MCRs have a sequence homology of 39 to 61% to one another at the amino acid level and rendering them able to bind to the natural melanocortin peptides at differential affinities. The core sequence, His-Phe-Arg-Trp, has been determined as the minimal active sequence for MCR activation [34]. In various cell types, binding ligands to MCRs leads to calcium fluxes, the activation of adenylate cyclase, and a resulting increase in intracellular cAMP, as well as the activation of MAPKs, which orchestrates an array of cellular functions [35,36]. Evidence from separate research endeavors, encompassing both expression and functional analyses, indicates that melanocortin receptors (MCRs) are expressed more extensively than initially perceived. In the eye, MCR-specific immunofluorescence was observed in Müller cells and ganglion cells in the retina, epithelial cells of the ciliary body, the iris pigment epithelium, and the cornea [37]. It is known that α-MSH can bind to rat intra-orbital and extra-orbital lacrimal gland membranes that express MCRs, but the specific subtypes of receptors are unknown [7]. It is worth noting that the expression of MCR isoforms can differ between species, and some cells may express multiple types of MCRs (Table 1). Both human choroidal and iridial melanocytes were found to express MC1R on their surface [38]. α-MSH has been shown to prevent the cytokine activation of nuclear factor-κB (NF-κB) in human uveal melanocytes [39]. But this finding has been challenged by other studies which have shown that uveal melanocytes do not express MCRs or respond to α-MSH [12,40]. Cheng et al. demonstrated that MC1R is functionally expressed in primary and transformed RPE cells. They demonstrated that α-MSH activated the Akt/mTOR and Erk1/2 MAPK signaling pathways in the RPE cells through MC1R and protected RPE cells from H_2_O_2_-induced apoptosis, an effect that was almost abolished when MC1R was depleted by siRNA [41]. Melanocortin receptor expression in the cornea, particularly MC1R, was investigated using a combined approach of fluorescent immunohistochemistry and Western blot analysis. This dual methodology confirmed robust MC1R expression in the primary human and murine corneal endothelium and cultured immortalized human (hCEnC-21T) and mouse (mCEnC-P2) endothelial cell lines [42,43]. Moreover, MC1R is expressed on various cells of the immune system, such as cytotoxic T cells and dendritic cells, where it can exert anti-inflammatory and immunomodulatory effects in the eye. α-MSH has been shown to induce tolerogenic dendritic cells capable of generating functional Tregs while suppressing the proliferation and cytokine secretion of pathogenic Th17 cells [44,45]. It has been reported that α-MSH suppresses antigen-induced B lymphocyte proliferation via MC1R and MC3R [46]. Of note, MC1R agonists have been proven to prevent ocular inflammation in preclinical disease models [47]. MC2R is a special member of MCRs that can only be activated by the adrenocorticotropic hormone [48], and thus would not be affected by peptide drugs based on α-MSH. In the retina, MC3R and MC4R are localized in the retinal ganglion-cell layer, while RPE cells abundantly express MC1R and MC5R [49]. Moreover, α-MSH acts predominantly on MC4R to antagonize hyperpermeability in retinal microvascular endothelial cells [50]. In this way, it inhibits blood–retinal barrier breakdown and vascular leakage and improves electrophysiological functions and morphology in early-diabetic retinas [50]. MC5R is required for APCs to modulate the immune response [51]. MC5R knockout mice did not experience the clinical benefits observed in wild-type mice when a gene therapy approach was employed to administer α-MSH into the eyes with uveitis. However, a histological examination indicated a reduction in damage and partial preservation of the retinal layers. Recent in vivo studies demonstrated that the activation of MC1R and MC5R exerts anti-angiogenic activity in the retina of diabetic mice [52,53]. MC1R and MC5R agonists reduced anti-inflammatory cytokines and chemokines and enhanced manganese superoxide dismutase and glutathione peroxidase levels of retinal cells, indicating that MC1R and MC5R agonists exert a protective role on experimental diabetic retinopathy [53]. In addition, blocking MC5R prevented α-MSH from suppressing IFN-γ production by the activated Tregs, suggesting that α-MSH-mediated immunoregulation is through the MC5R on primed T cells [30].

## 3. Biological Functions of Melanocortin Stimulation

### 3.1. Cytoprotective Effects of α-MSH

**Figure 1:**
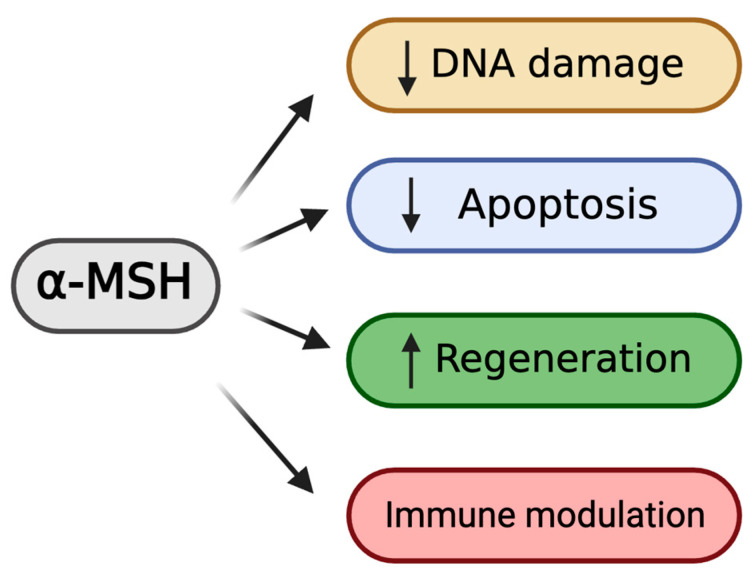
Biological functions of melanocortin stimulation.

α-melanocyte-stimulating hormone (α-MSH) manifests protective efficacy against cellular toxicity and apoptosis-inducing signals (Figure 1). The targeted delivery of α-MSH has been observed to potentiate reparative processes following nerve lesions in rats [54]. In the rat weight drop injury model, treatment with subcutaneous α-MSH after trauma led to a substantial salvage of spinal cord function [55]. Earlier studies focused on the cyto-protective effect of melanocortin receptor activation in neuronal cells. In recent studies, the functional aspects of α-MSH have been identified as a potent regulator of apoptosis triggered by genotoxic stress. In various cutaneous cells exposed to the environmental stressor ultraviolet radiation B (UV-B), the administration of α-MSH resulted in a noteworthy inhibition of apoptosis [56]. The anti-apoptotic impact of α-MSH can be triggered by doses ranging from nanomolar to micromolar concentrations of the peptide, resulting in a decrease in the levels of DNA photoproducts [56,57]. The implicated cyto-protective mechanism is via α-MSH’s suppressive capacity on UVB-induced H_2_O_2_-, IL-1β-, and TNF-α-related cell death [57,58]. α-MSH has the capability to suppress reactive oxygen species (ROS), as evidenced by its ability to impede the generation of superoxide radicals in rat neutrophils supplemented with LPS or phorbol ester [59]. The generation of inducible nitric oxide synthase (iNOS) and the subsequent release of the potent vasodilator nitric oxide (NO) as a result of cellular exposure to proinflammatory stressors can also be suppressed by α-MSH [60,61,62,63,64,65,66]. In cold-stored donor corneas, our group performed an ex-vivo study subjecting tissues to oxidative stress from hydrogen peroxide or cytokine-induced stress from TNF-α and IFN-γ. This study showed that the retained CEnC number was significantly higher in the α-MSH treated group, demonstrating the cytoprotective effect of α-MSH on CEnCs [67]. In addition, our group recently demonstrated α-MSH’s ability to rescue human CEnCs in an in vitro oxidative challenge. The treatment resulted in a reduction the number of DNA double-strand breaks and the attenuation of cell apoptosis and necrosis in CEnCs challenged with H_2_O_2_ [68]. Moreover, we reported that subconjunctival supplementation with α-MSH following acute trans-corneal freezing effectively suppresses CEnC apoptosis [43]. Another study showed that α-MSH protects human corneal epithelial cells (hCECs) by preserving their (1) viability and (2) migratory capacity, and by decreasing apoptosis via the epithelial growth factor receptor (EGFR) through the JAK-STAT signaling pathway [69].

Delving into the molecular mechanisms through which α-MSH and MCRs suppress apoptosis in neuronal and associated cell types, in the immortalized hypothalamic tumor cell line, GT1-1, the synthetic analog of α-MSH, NDP-MSH, at a dose of 10^−6^ M, was shown to inhibit serum deprivation-induced caspase 3 activation. This anti-apoptotic effect of NDP-MSH is mainly mediated through the activation of the ERK1/2 pathway via binding to MC4R [70]. In another report, α-MSH-reduced IFN-γ/LPS lead to apoptosis of rat astrocytes by shifting the expression of the apoptotic modulators Bax/Bcl2 [65]. α-MSH mitigated the cyclosporine-induced upregulation of the proapoptotic regulator Bax, elevated the expression of the antiapoptotic Bcl2, and diminished TGF-β levels in the kidney [71]. In a murine model of renal ischemia–reperfusion, an intravenous delivery of 25 μg of α-MSH was demonstrated to not only alleviate the activation of p38 and NF-κB, as well as the DNA binding of activator protein 1 (AP1) in the kidney, but also displayed similar protective effects in distant tissues such as the lung [72].

### 3.2. Pro-Regenerative Effects of α-MSH

A growing body of research has demonstrated the ability of α-MSH to promote regeneration and wound healing in corneal tissues through several mechanisms. A scratch assay for cultured human CEnC in vitro showed that supplementation with α-MSH at nanomolar concentrations markedly accelerates the closure of scratch wounds, indicating enhanced directional migration [43]. Additionally, α-MSH induces a dose-dependent proliferation of CEnCs [43]. The pro-regenerative effects of α-MSH have also been confirmed in vivo using animal models of acute corneal injury. Following trans-corneal freezing, treatment with α-MSH significantly increases the frequency of proliferating CEnCs in the periphery of the wound and accelerates the repopulation of the damaged endothelial layer compared to untreated controls [43]. The ability of α-MSH to stimulate both the migration and proliferation of corneal endothelial cells suggests direct mitogenic mechanisms that facilitate swift recovery after insults to the endothelial layer. Overall, these findings support the role of melanocortin receptor activation in the promotion of corneal wound healing and endothelial regeneration after acute injuries or surgery.

### 3.3. Anti-Inflammatory Effects of α-MSH

α-MSH has demonstrated an array of anti-inflammatory functions in a multitude of cell types and animal models. Initial investigations into the anti-inflammatory properties of α-MSH at the cellular level concentrated on its suppressive impact on the expression of proinflammatory cytokines, specifically interferon gamma (IFN-γ) and tumor necrosis factor alpha (TNF-α). Taylor et al. identified the expression of α-MSH within the aqueous humor and observed its inhibitory effect on IFN-γ production by antigen-stimulated primed lymph node T cells [20,73]. Lipopolysaccharide (LPS)-induced TNF-α expression in monocytes/macrophages could be reduced by α-MSH with doses from 10^−16^ to 10^−12^ M. Expression of proinflammatory cytokines IL-1, IL-6, and IL-8 has also been shown to be regulated by α-MSH [74,75]. α-MSH treatment also leads to the downregulation of IL-8 receptors in human neutrophils [76]. In addition to inhibiting proinflammatory cytokines, α-MSH has shown strong suppression of the expression of intercellular adhesion molecule 1 (ICAM-1) induced by IFN-γ, LPS, or TNF-α [77,78,79,80,81]. The co-stimulatory molecules CD86 and CD40, crucial for antigen presentation by monocytes and dendritic cells, undergo modulation by α-MSH [82]. Furthermore, α-MSH demonstrated the ability to induce tolerogenic dendritic cells, capable of expanding Tregs in vitro, as well as in vivo in an skin inflammation model [45]. However, α-MSH was reported to have no demonstrable effect on the maturation of in vitro expanded murine dendritic cells [83].

The anti-inflammatory effect of α-MSH has been studied in numerous diseases in different systems [84,85,86]. In a model of endotoxin-induced uveitis, an intravenous administration of α-MSH resulted in a dose-dependent reduction in the number of infiltrating cells in the anterior chamber, along with a significant reduction in tumor necrosis factor alpha (TNF-α), interleukin 6 (IL-6), nitric oxide (NO), macrophage inhibitory protein 2, and monocyte chemoattractant protein 1 (MCP-1) in the aqueous humor [29]. Not only was it shown to suppress proinflammatory mediators, α-MSH was also shown to increase the expression of IL-10, which possesses potent immunosuppressive activities. The stimulation of peripheral blood mononuclear cells (PBMCs) with α-MSH at concentrations of 10^−10^ and 10^−12^ M increased the mRNA and protein expression of IL-10 [87]. Analogous inductive effects were described in human epidermal keratinocytes [88]. In the eye, topical α-MSH eye drops upregulated IL-10 expression at the transcriptomic level in the trigeminal ganglia and corneas of a suture-induced corneal neovascularization mouse model [89].

While the administration of α-MSH inhibited both the sensitization and induction phases of the cutaneous immune response, the induction of in vivo tolerance by α-MSH could be nullified by the application of an antibody targeting IL-10. This strongly indicates that IL-10 serves as a pivotal component in the molecular mechanism through which α-MSH exerts its anti-inflammatory effects [90,91]. In regard to the cornea, subconjunctival injections of α-MSH were shown to significantly reduce the infiltration of neutrophils and macrophages into the corneal tissue following trans-corneal freezing [43]. Correspondingly, a similar mode and dosage of α-MSH therapy decreased neutrophil infiltration into the graft after corneal transplantation in mice [92].

α-MSH also modulates lymphocyte activity and proliferation. The heightened induction of regulatory T cells (Tregs) was particularly notable when previously sensitized T cells were activated in vitro with the inclusion of α-MSH, followed by subsequent exposure to TGF-β [29]. The Tregs induced by α-MSH demonstrated the expression of CD25 and CD4 markers while concurrently inhibiting the production of IFN-γ by effector T cells in vitro. In the context of corneal transplantation, a twice-weekly subconjunctival injection of α-MSH at a 10^−4^ M concentration enhanced the expression of FoxP3 in Tregs and increased the frequencies of IL-10^+^ and TGF-β^+^ Tregs [42]. The subconjunctival injections also decreased the frequency of IFN-γ^+^ T cells, which have been shown to contribute to corneal allograft rejection [93]. Similarly, the genomic expression of Th1-associated cytokines, IL-2 and IFN-γ, was reduced in transplanted animals that received α-MSH subconjunctival injections [92]. Cooper et al. demonstrated that α-MSH suppresses the proliferation of human T lymphocytes stimulated with the bacterial antigens streptokinase/streptodornase [46]. Additionally, the production of α-MSH is required for the modulation of macrophage and microglial cell activity [94].

The inhibition of NF-κB is suggested as a fundamental molecular mechanism contributing to the anti-inflammatory effects of α-MSH, particularly in its ability to modulate the expression of proinflammatory cytokines and adhesion molecules. It was reported that α-MSH at nanomolar doses inhibited the activation of NF-κB in response to TNF-α, IL-1, and LPS [76]. This observation was confirmed in a number of cell types using various proinflammatory stimuli [76,77,78,79]. In terms of mechanisms, α-MSH activates NF-κB through elevated cAMP levels, and this is associated with the prevention of the degradation of the inhibitory subunit of NF-κB, IκBα. Consequently, the translocation of the p65 subunit of NF-κB to the nucleus is inhibited [95,96,97,98]. It was demonstrated that hydrogen peroxide (H_2_O_2_) induced NF-κB activation, a response that could be averted by 100 nM of α-MSH in the rat small intestine cell line IEC-6 [99]. Notably, α-MSH exhibited the capability to reverse the H_2_O_2_-induced inhibition of scratch wounding in IEC-6 cells, indicating a potential role of melanocortin peptides in promoting epithelial restitution. Consistent with its inhibitory impact on NF-κB activation and cytokine production in vitro, α-MSH maintained the expression of IκBα protein and reduced TNF-α expression in the murine brain following the injection of mice with LPS [5,98].

### 3.4. Anti-Angiogenic Effects of α-MSH

Corneal inflammation triggers angiogenesis to supply necessary nutrients and oxygen to the affected area. However, an imbalance in this process can lead to excessive blood vessel growth, resulting in corneal neovascularization, potentially impairing vision. A healthy cornea typically maintains its transparency and clarity by being avascular, devoid of any visible blood vessels. Corneal angiogenic privilege is the result of the complex interplay of neuropeptides, cytokines, and other biological factors [100]. The contributions of α-MSH to the maintenance of corneal transparency was first described by Bock et al. in 2016. Topical application of aqueous humor was demonstrated to prevent hemangiogenesis and lymphangiogenesis in sutured murine corneas. Moreover, in vitro studies confirmed the antiangiogenic properties of the aqueous humor via suppression of human lymphatic and vascular endothelial cell proliferation. These effects were further shown to be partially mediated by α-MSH through aqueous humor depletion studies [10,101]. In one study, cultured human umbilical vein endothelial cells (HUVECs), rat aorta rings, and transgenic zebrafish were utilized to investigate the mechanism through which α-MSH inhibits neovascularization. The study found that α-MSH attenuates neovascularization by inducing NO deficiency through the MCR/protein kinase A (PKA)/NF-κB signaling pathway and inhibits physiological angiogenesis by attenuating VEGF/VEGFR2/Akt signaling [102]. More recently, Yin et al. shed light on the source of α-MSH and delineated its anti-hemangiogenic and anti-lymphangiogenic potential. Their studies reported α-MSH’s expression by corneal nerves rooted in the trigeminal ganglion (TG) [103]. Both murine vascular endothelial cells co-cultured with TG or cultured with TG conditioned media exhibited decreased proliferation [103,104], and α-MSH was observed to reduce the proliferation, migration, and tube formation of murine vascular endothelial cells and human retina endothelial cells in vitro [89,104]. Studies with the RNAi inhibition of α-MSH in the TG corroborated the previous findings [104]. Similarly, suture-induced in vivo angiogenesis in a mouse cornea was reduced through a topical treatment with TG-conditioned media and α-MSH at a 10^−4^ M concentration [89,104]. The specificity was confirmed in vivo through studies antagonizing α-MSH signaling with agouti-signaling protein [104]. Lastly, the treatment with α-MSH decreased VEGF-A and pro-inflammatory cytokine (such as IL-6 and TNF) expression at the mRNA level in sutured murine corneas [89]. While there is still much to be investigated, these studies open up an intriguing avenue of research by revealing the pivotal role of α-MSH in regulating corneal angiogenesis.

## 4. Application of α-MSH in Treating Eye Disease (Figure 2 and Table 2)

### 4.1. α-MSH Application in the Cornea

The ocular surface system comprises the cornea, conjunctiva, lacrimal glands, accessory lacrimal glands, meibomian glands, lacrimal drainage apparatus, eyelids, and interconnected nerves. It has a central role in protecting the rest of the eye and preserving the refractive properties of the cornea, and, hence, vision [105]. The optimal function of corneal nerves is essential for the maintenance of a healthy ocular surface. The corneal epithelium possesses around 7000 nerve endings per square millimeter, rendering the cornea the most densely innervated tissue in the human body [106]. It is thus not surprising that neuropeptides (produced and released by neurons) play an important role in regulating ocular surface homeostasis. The immunomodulatory properties of α-MSH have suggested that it may be an effective and safe immunosuppressive therapy.

**Table 2:** Applications of α-MSH in the treatment of ocular diseases.

	Target Tissue	Mechanism of Action	Observed Function
Maintenance of tear production and corneal function	Lacrimal gland; Corneal epithelium and endothelium (CEnC).	Protein phosphorylation through the activation of a cAMP-dependent pathway in lacrimal gland; Modulation of EGFR in corneal epithelium; Suppression of pro-inflammatory cytokines (TNF-α, IL-1β, and IFN-γ) at the ocular surface; Suppression of delayed-type hypersensitivity and gene expression of IFN-γ and IL-2.	Amelioration of tear secretion, tear film stability, and corneal integrity; Suppression of ocular surface inflammation; Maintenance of corneal integrity; Enhancement of CEnC in corneal graft survival.
Acceleration of corneal wound healing	Human corneal tissue.	Stimulation of nitric oxide (NO) disposition; Inhibition of apoptosis pathways.	Promotion of corneal wound healing; Reduction in local inflammatory response; Prevention of corneal edema and opacity; Suppression of CEnC apoptosis while promoting proliferation; Long-term protective effect.
Regulation of Allergic Conjunctivitis	Peripheral blood.	Suppression of CD203c upregulation; Suppression of eosinophilic chemoattractant factor release from Th2 cells and airway epithelial cells; Reduction in systemic levels of IL-6 and IL-4; Restoring of Treg frequency and downregulation of CD4^+^ effector cells activation.	Regulation of allergic conjunctivitis immune response.
Prevention of conjunctival fibrosis	Human Tenon’s capsule cells.	Suppression of fibroblast proliferation; Modulation of TGF-β1-dependent collagen gene expression.	Potential treatment for conjunctival fibrosis.
Cyto-protection of uveal and retinal tissues	Experimental autoimmune uveitis (EAU) model; Retinal transplantation model; Retinal dystrophy model; Diabetic retinopathy.	Suppression of cyclooxygenase-2 production by macrophages in EAU; Prevention of infiltration of immune cell inflammation and production of proinflammatory cytokines and chemokines in the eye following EAU; Activation of MC1R and MC5R in EAU; α-MSH-generated interphotoreceptor retinoid-binding protein (IRBP)-specific Treg cells in retinal transplantation model; Protect RPE cells from H_2_O_2_-induced apoptosis through MC1R activation; Protective effects in retinal vascular endothelial cells mediated by the inhibition of Foxo4 upregulation; Stimulation of PGE2 and prostacyclin levels in ciliary body and iris cells.	Acceleration of recovery, suppresses severity, and hastens resolution in uveitis; Promotion retinal allograft survival and development; Protection RPE cells from apoptosis; Normalization of oxidative stress in retina; Prevention of retinal vascular endothelial cells dysfunction in diabetes; Control of intraocular pressure.

It has been reported that MC5R mRNA was found in the lacrimal gland, indicating that α-MSH influences the physiological functions of the lacrimal gland, which is crucial in dry eyes [107]. Histologically, α-MSH was found to be expressed primarily at the basal perinuclear region within the acinar cells [7]. Functionally, α-MSH has been shown to induce tear secretion and protein phosphorylation in the rat lacrimal gland through the activation of a cAMP-dependent pathway [108]. Ru et al. administered α-MSH twice a day to the ocular surface in a dry-eye model and showed that α-MSH at various doses improved tear secretion, enhanced tear film stability, preserved corneal integrity, and suppressed the overexpression of proinflammatory factors TNF-α, IL-1β, and IFN-γ on the ocular surface [8]. Moreover, they showed that α-MSH, at a concentration of 10^−4^ μg/μL, maintained corneal morphology, inhibited cell apoptosis, and restored both the number and the phenotype of conjunctival goblet cells. Another research team independently observed comparable treatment outcomes of α-MSH in the same dry-eye model and determined that these protective effects were mediated through EGFR [69]. Following the positive outcomes and minimal adverse effects of treatment with a pan-melanocortin (except MCR2) agonist in a phase 2 clinical trial of dry eye disease, a phase 3 trial, MELODY-1, is now in progress [109].

Our lab demonstrated that a significant increase in corneal graft survival can be achieved with subconjunctival injections of α-MSH twice weekly in a murine orthotopic corneal transplantation model [42]. These findings indicate that α-MSH could potentially enhance the survival of corneal endothelial cells (CEnCs) following transplantation and provide protection to the endothelium against proinflammatory cytokines and oxidative stress. This is especially significant for patients at an elevated risk of graft complications [67]. α-MSH exhibited a reduction in the leukocyte count within a cornea graft. Moreover, in α-MSH-treated mice, there was a notable decrease in allo-specific delayed-type hypersensitivity, along with a significant reduction in the gene expression of IFN-γ and IL-2, as compared to the control group [92].

It has been found that topical α-MSH treatment could accelerate the rate of corneal wound healing and reduce the local inflammatory response in rats [110]. Similar anti-inflammatory effectiveness was observed whether α-MSH was administered systemically or topically to the eyes of rabbits undergoing surgical trauma to the cornea [111]. Topical administration of the COOH-terminal tripeptide sequence of α-MSH may facilitate rabbit corneal epithelial wound healing through a mechanism that may involve nitric oxide (NO) availability in corneal tissue. Regarding the conjunctiva, human Tenon’s capsule fibroblasts (HTFs) play a crucial role as the primary effector cells initiating wound healing and fibrotic scar formation following trabeculectomy. Research has indicated that α-MSH has the capacity to efficiently inhibit HTF proliferation and regulate pertinent genes involved in collagen synthesis stimulated by TGF-β1. This suggests that α-MSH might be a potential treatment option for conjunctival fibrotic scar disorders [112]. Recently, we applied α-MSH on a trans-corneal injury model and found that it prevented corneal edema and opacity, reduced leukocyte infiltration, and limited CEnC apoptosis while promoting their proliferation [43]. Meanwhile, α-MSH exhibited long-term protective effects against UV-induced phenotypic corneal endothelial changes in mice [68].

With the growing body of evidence demonstrating the role of MC1R regulation in the treatment of allergic rhinitis and asthma, the effect of α-MSH supplementation in immunological regulation in allergic conjunctivitis has been recently explored [113,114]. Kleiner et al. demonstrated that, upon allergen exposure, patients with allergic rhinitis had an accumulation of MC1R-positive basophils in the nasal mucosa. In vitro, the upregulation of CD203c by anti-IgE was suppressed upon α-MSH supplementation [113]. Webering et al. showed that the neutralization of α-MSH in a murine model of asthma led to increased mucus production and airway inflammation. A supplementation of α-MSH led to a suppression of the release of eosinophilic chemoattractant factor from Th2 and airway epithelial cells [114]. When α-MSH was added to the peripheral blood mononuclear cells (PBMCs) of patients with perennial allergic conjunctivitis (PAC), the concentrations of IL-6 and IL-4 were diminished, the frequency of Tregs was increased, and CD4 activation was downregulated [102]. Cheng et al. employed primary cultures of orbital fibroblasts derived from individuals with thyroid eye disease (TED) and highlighted substantial anti-inflammatory attributes of α-MSH. This suggests a possible involvement of α-MSH in modulating the immune response in TED [102].

### 4.2. α-MSH Application in the Uvea and Retina

In addition to addressing ocular surface diseases, α-MSH has been utilized in the treatment of experimental autoimmune uveitis (EAU) and endotoxin-induced uveitis (EIU) to assess its effectiveness in preventing, suppressing, and restoring ocular immune privilege. It was found that a systemic injection of α-MSH in EAU accelerated the recovery of the disease [13]. Subconjunctival injections of α-MSH at the onset of inflammation suppressed the severity and hastened the resolution of the disease [115]. The mechanism underlying the suppression of endotoxin-induced uveitis (EIU) by α-MSH was elucidated through the inhibition of cyclooxygenase-2 production by macrophages in the ocular microenvironment. This inhibition prevented the subsequent infiltration of immune cells and the production of proinflammatory cytokines and chemokines in the eye [116,117]. In a retinal transplantation model, an adoptive transfer of α-MSH-generated interphotoreceptor retinoid-binding protein (IRBP)-specific Treg cells was shown to promote retinal allograft survival and development [118]. Another study recently discovered that the therapeutic effect of α-MSH in EAU was primarily achieved by acting on MC1R and MC5R [14].

The prospective therapeutic benefits associated with α-MSH extend beyond ocular inflammatory conditions to encompass a spectrum of ocular pathologies. α-MSH treatment has demonstrated efficacy in stimulating neurite outgrowth from embryonic retinal explants. Furthermore, during the developmental stages of chick embryonic eyes, a discernible spatial and temporal expression of α-MSH has been identified specifically within the cells of the retinal pigment epithelium (RPE) [49,119]. These findings suggest that in addition to its previously described immunomodulatory activity, α-MSH also functions as a neurotropic factor that may play a crucial role in the development and survival of the retina. α-MSH was found to protect RPE cells from H_2_O_2_-induced apoptosis, an effect that was almost abolished when MC1R was knocked down by siRNA [41]. It has been reported that intravitreal injections of α-MSH analogs retard photoreceptor loss in retinal dystrophy rats [120]. In early-diabetic retinas, α-MSH normalized oxidative stress, reduced apoptosis and ultrastructural injuries, and corrected gene expression levels. The protective effects of α-MSH in retinal vascular endothelial cells may be mediated through the inhibition of Foxo4 upregulation induced by high glucose [9]. There is evidence supporting the effectiveness of topically applied α-MSH in reducing intraocular pressure in rabbits with normal tension for a duration of up to six hours. This effect is achieved by stimulating the levels of PGE2 and prostacyclin in cells of the iris and ciliary body [120]. This finding suggests a possibility of utilizing α-MSH as a promising anti-glaucoma medication.

**Figure 2:**
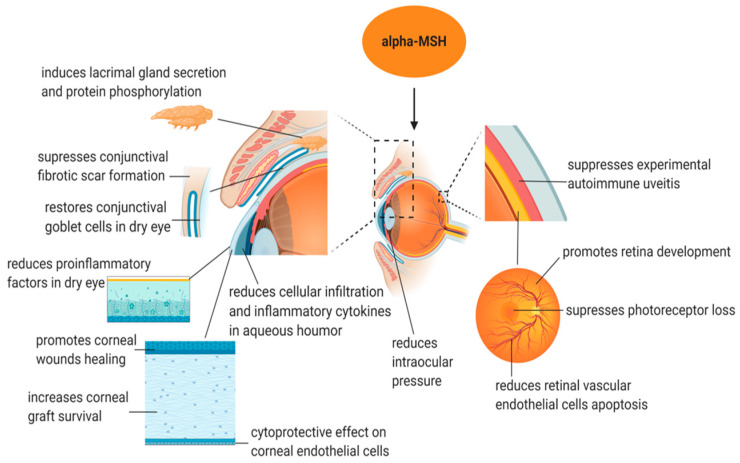
Potential therapeutic applications of α-MSH in eye conditions.

## 5. Conclusions

Significant strides have been achieved in unraveling the multifaceted roles of α-MSH across various tissues and organs, yet a notable gap persists in our understanding of its specific biological functions within the eye, particularly in the cornea and ocular surface. Recent advancements have, however, contributed valuable insights into the therapeutic potential of α-MSH in ocular health. The evolving landscape of research underscores the imperative for further investigations to comprehensively elucidate the intricate molecular mechanisms governing α-MSH’s diverse effects on corneal cells, endothelial tissues, and inflammatory pathways.

A critical frontier in this pursuit involves the identification of precise melanocortin receptor subtypes responsible for mediating α-MSH responses within ocular tissues. This knowledge will not only deepen our understanding of the intricate interplay at the molecular level but also pave the way for targeted therapeutic interventions. Additionally, a rigorous exploration of the pharmacokinetics and bioavailability of α-MSH in ocular microenvironments is paramount. Such investigations will inform optimal dosage regimens and delivery methods, ensuring the precision and efficacy of α-MSH-based interventions for a spectrum of ocular diseases.

In conclusion, while the current body of research marks substantial progress, the journey toward harnessing the full therapeutic potential of α-MSH in ocular health requires ongoing dedication to unraveling its complexities. Further studies are warranted to bridge existing knowledge gaps, ultimately fostering the development of tailored and effective therapeutic approaches for a diverse array of ocular conditions.

## Figures and Tables

**Table 1:** Expression and function of α-MSH receptors in the eye.

Receptor	Site of Expression	Functions
MC1R	Uveal Melanocytes	Inhibits cytokine-stimulated NF-κB activity
	Corneal Endothelial Cells	Cytoprotection Regeneration
	RPE	Inhibits apoptosis
	T cells	Expands Tregs and suppresses Th17 proliferation
	B lymphocytes	Suppresses antigen-induced proliferation
MC3R	B lymphocytes	Suppress antigen-induced lymphocyte proliferation
	Retinal ganglion cells	Stimulates neurite growth from retinal neurons
MC4R	Retinal micro-vessel endothelial cells	Antagonizes hyperpermeability
	Retinal ganglion cells	Stimulates neurite growth from retinal neurons
MC5R	RPE cells	Mitigates the release of cytokines and angiogenesis
	Primed T cells	Suppresses INF-γ by activating Treg cells

## References

[1] Cooray S.N., Clark A.J.L. (2011). Melanocortin Receptors and Their Accessory Proteins. Mol. Cell Endocrinol..

[2] Ceriani G., Diaz J., Murphree S., Catania A., Lipton J.M. (1994). The Neuropeptide Alpha-Melanocyte-Stimulating Hormone Inhibits Experimental Arthritis in Rats. Neuroimmunomodulation.

[3] Guarini S., Bazzani C., Bertolini A. (1997). Resuscitating Effect of Melanocortin Peptides after Prolonged Respiratory Arrest. Br. J. Pharmacol..

[4] Lipton J.M., Ceriani G., Macaluso A., McCoy D., Carnes K., Biltz J., Catania A. (1994). Antiinflammatory Effects of the Neuropeptide α-MSH in Acute, Chronic, and Systemic Inflammation. Annals of the New York Academy of Sciences.

[5] Rajora N., Boccoli G., Burns D., Sharma S., Catania A.P., Lipton J.M. (1997). α-MSH Modulates Local and Circulating Tumor Necrosis Factor-α in Experimental Brain Inflammation. J. Neurosci..

[6] Rajora N., Boccoli G., Catania A., Lipton J.M. (1997). α-MSH Modulates Experimental Inflammatory Bowel Disease. Peptides.

[7] Leiba H., Garty N.B., Schmidt-Sole J., Piterman O., Azrad A., Salomon Y. (1990). The Melanocortin Receptor in the Rat Lacrimal Gland: A Model System for the Study of MSH (Melanocyte Stimulating Hormone) as a Potential Neurotransmitter. Eur. J. Pharmacol..

[8] Ru Y., Huang Y., Liu H., Du J., Meng Z., Dou Z., Liu X., Wei R.H., Zhang Y., Zhao S. (2015). α-Melanocyte-Stimulating Hormone Ameliorates Ocular Surface Dysfunctions and Lesions in a Scopolamine-Induced Dry Eye Model via PKA-CREB and MEK-Erk Pathways. Sci. Rep..

[9] Zhang L., Dong L., Liu X., Jiang Y., Zhang L., Zhang X., Li X., Zhang Y. (2014). A-Melanocyte-Stimulating Hormone Protects Retinal Vascular Endothelial Cells From Oxidative Stress and Apoptosis in a Rat Model of Diabetes. PLoS ONE.

[10] Bock F., Onderka J., Braun G., Schneider A.C., Hos D., Bi Y., Bachmann B.O., Cursiefen C. (2016). Identification of Novel Endogenous Anti(Lymph)Angiogenic Factors in the Aqueous Humor. Investig. Ophthalmol. Vis. Sci..

[11] Sanjiv N., Osathanugrah P., Fraser E., Ng T.F., Taylor A.W. (2021). Extracellular Soluble Membranes from Retinal Pigment Epithelial Cells Mediate Apoptosis in Macrophages. Cells.

[12] Li L., Hu D.N., Zhao H., McCormick S.A., Nordlund J.J., Boissy R.E. (2006). Uveal Melanocytes Do Not Respond to or Express Receptors for α-Melanocyte-Stimulating Hormone. Investig. Ophthalmol. Vis. Sci..

[13] Taylor A.W., Yee D.G., Nishida T., Namba K. (2000). Neuropeptide Regulation of Immunity: The Immunosuppressive Activity of Alpha-Melanocyte-Stimulating Hormone (α-MSH). Annals of the New York Academy of Sciences.

[14] Ng T.F., Dawit K., Taylor A.W. (2022). Melanocortin Receptor Agonists Suppress Experimental Autoimmune Uveitis. Exp. Eye Res..

[15] Taylor A.W., Lee D. (2010). Applications of the Role of α-MSH in Ocular Immune Privilege. Adv. Exp. Med. Biol..

[16] Ferguson T.A., Griffith T.S. (2007). The Role of Fas Ligand and TNF-Related Apoptosis-Inducing Ligand (TRAIL) in the Ocular Immune Response. Chem. Immunol. Allergy.

[17] Niederkorn J.Y. (2003). The Immune Privilege of Corneal Grafts. J. Leukoc. Biol..

[18] Taylor A.W. (2003). A Review of the Influence of Aqueous Humor on Immunity. Ocul. Immunol. Inflamm..

[19] Streilein J.W., Masli S., Takeuchi M., Kezuka T. (2002). The Eye’s View of Antigen Presentation. Hum. Immunol..

[20] Taylor A.W., Streilein J.W., Cousins S.W. (1992). Identification of Alpha-Melanocyte Stimulating Hormone as a Potential Immunosuppressive Factor in Aqueous Humor. Curr. Eye Res..

[21] Ding W., Stohl L.L., Wagner J.A., Granstein R.D. (2008). Calcitonin Gene-Related Peptide Biases Langerhans Cells toward Th2-Type Immunity. J. Immunol..

[22] Delgado M., Pozo D., Ganea D. (2004). The Significance of Vasoactive Intestinal Peptide in Immunomodulation. Pharmacol. Rev..

[23] Niizeki H., Alard P., Streilein J.W. (1997). Calcitonin Gene-Related Peptide Is Necessary for Ultraviolet B-Impaired Induction of Contact Hypersensitivity. J. Immunol..

[24] Sung C.P., Arleth A.J., Aiyar N., Bhatnagar P.K., Lysko P.G., Feuerstein G. (1992). CGRP Stimulates the Adhesion of Leukocytes to Vascular Endothelial Cells. Peptides.

[25] Columbo M., Horowitz E.M., Kagey-Sobotka A., Lichtenstein L.M. (1996). Substance P Activates the Release of Histamine from Human Skin Mast Cells through a Pertussis Toxin-Sensitive and Protein Kinase C-Dependent Mechanism. Clin. Immunol. Immunopathol..

[26] Sun J., Ramnath R.D., Zhi L., Tamizhselvi R., Bhatia M. (2008). Substance P Enhances NF-ΚB Transactivation and Chemokine Response in Murine Macrophages via ERK1/2 and P38 MAPK Signaling Pathways. Am. J. Physiol. Cell Physiol..

[27] Phan T.A., Taylor A.W. (2013). The Neuropeptides α-MSH and NPY Modulate Phagocytosis and Phagolysosome Activation in RAW 264.7 Cells. J. Neuroimmunol..

[28] Gonzalez-Rey E., Ganea D., Delgado M. (2010). Neuropeptides: Keeping the Balance between Pathogen Immunity and Immune Tolerance. Curr. Opin. Pharmacol..

[29] Nishida T., Taylor A.W. (1999). Specific Aqueous Humor Factors Induce Activation of Regulatory T Cells. Investig. Ophthalmol. Vis. Sci..

[30] Taylor A.W., Namba K. (2001). In Vitro Induction of CD25+ CD4+ Regulatory T Cells by the Neuropeptide Alpha-Melanocyte Stimulating Hormone (α-MSH). Immunol. Cell Biol..

[31] Namba K., Kitaichi N., Nishida T., Taylor A.W. (2002). Induction of Regulatory T Cells by the Immunomodulating Cytokines α-Melanocyte-Stimulating Hormone and Transforming Growth Factor-Β2. J. Leukoc. Biol..

[32] Taylor A.W. (2003). Modulation of Regulatory T Cell Immunity by the Neuropeptide Alpha-Melanocyte Stimulating Hormone. Cell. Mol. Biol..

[33] Nieto J.E., Casanova I., Serna-Ojeda J.C., Graue-Hernández E.O., Quintana G., Salazar A., Jiménez-Martinez M.C. (2020). Increased Expression of Tlr4 in Circulating Cd4+t Cells in Patients with Allergic Conjunctivitis and in Vitro Attenuation of Th2 Inflammatory Response by Alpha-Msh. Int. J. Mol. Sci..

[34] Hruby V.J., Wilkes B.C., Hadley M.E., Al-obeidi F., Sawyer T.K., Staples D.J., Devaux A.E., Dym O., Castrucci A.M.d.L., Hintz M.F. (1987). α-Melanotropin: The Minimal Active Sequence in the Frog Skin Bioassay. J. Med. Chem..

[35] Elliott R.J., Szabo M., Wagner M.J., Kemp E.H., MacNeil S., Haycock J.W. (2004). α-Melanocyte-Stimulating Hormone, MSH 11-13 KPV and Adrenocorticotropic Hormone Signalling in Human Keratinocyte Cells. J. Investig. Dermatol..

[36] Englaro W., Rezzonico R., Durand-Clément M., Lallemand D., Ortonne J.P., Ballotti R. (1995). Mitogen-Activated Protein Kinase Pathway and AP-1 Are Activated during CAMP-Induced Melanogenesis in B-16 Melanoma Cells. J. Biol. Chem..

[37] Mirshahi M., Mirshahi A., Sedighian R., Hecquet C., Faure J.P., Agarwal M.K. (1997). Immunochemical Demonstration of the Mineralocorticoid Receptor in Ocular Tissues. Neuroendocrinology.

[38] Smith-Thomas L.C., Moustafa M., Dawson R.A., Wagner M., Balafa C., Haycock J.W., Krauss A.H.P., Woodward D.F., Macneil S. (2001). Cellular and Hormonal Regulation of Pigmentation in Human Ocular Melanocytes. Pigment Cell Res..

[39] Haycock J.W., Wagner M., Morandini R., Ghanem G., Rennie I.G., Macneil S. (1999). α-MSH Immunomodulation Acts via Rel/NF-ΚB in Cutaneous and Ocular Melanocytes and in Melanoma Cells. Annals of the New York Academy of Sciences.

[40] Hintermann E., Erb C., Talke-Messerer C., Liu R., Tanner H., Flammer J., Eberle A.N. (2001). Expression of the Melanin-Concentrating Hormone Receptor in Porcine and Human Ciliary Epithelial Cells. Investig. Ophthalmol. Vis. Sci..

[41] Cheng L.B., Cheng L., Bi H.E., Zhang Z.Q., Yao J., Zhou X.Z., Jiang Q. (2014). Alpha-Melanocyte Stimulating Hormone Protects Retinal Pigment Epithelium Cells from Oxidative Stress through Activation of Melanocortin 1 Receptor-Akt-MTOR Signaling. Biochem. Biophys. Res. Commun..

[42] Lužnik Marzidovšek Z., Blanco T., Sun Z., Alemi H., Ortiz G., Nakagawa H., Chauhan S.K., Taylor A.W., Jurkunas U.V., Yin J. (2022). The Neuropeptide Alpha-Melanocyte–Stimulating Hormone Is Critical for Corneal Endothelial Cell Protection and Graft Survival after Transplantation. Am. J. Pathol..

[43] Alemi H., Wang S., Blanco T., Kahale F., Singh R.B., Ortiz G., Musayeva A., Yuksel E., Pang K., Deshpande N. (2024). The Neuropeptide α-Melanocyte–Stimulating Hormone Prevents Persistent Corneal Edema Following Injury. Am. J. Pathol..

[44] Loser K., Brzoska T., Oji V., Auriemma M., Voskort M., Kupas V., Klenner L., Mensing C., Hauschild A., Beissert S. (2010). The Neuropeptide Alpha-Melanocyte-Stimulating Hormone Is Critically Involved in the Development of Cytotoxic CD8+ T Cells in Mice and Humans. PLoS ONE.

[45] Auriemma M., Brzoska T., Klenner L., Kupas V., Goerge T., Voskort M., Zhao Z., Sparwasser T., Luger T.A., Loser K. (2012). α-MSH-Stimulated Tolerogenic Dendritic Cells Induce Functional Regulatory T Cells and Ameliorate Ongoing Skin Inflammation. J. Investig. Dermatol..

[46] Cooper A., Robinson S.J., Pickard C., Jackson C.L., Friedmann P.S., Healy E. (2005). α-Melanocyte-Stimulating Hormone Suppresses Antigen-Induced Lymphocyte Proliferation in Humans Independently of Melanocortin 1 Receptor Gene Status. J. Immunol..

[47] Spana C., Taylor A.W., Yee D.G., Makhlina M., Yang W., Dodd J. (2019). Probing the Role of Melanocortin Type 1 Receptor Agonists in Diverse Immunological Diseases. Front. Pharmacol..

[48] Fridmanis D., Roga A., Klovins J. (2017). ACTH Receptor (MC2R) Specificity: What Do We Know about Underlying Molecular Mechanisms?. Front. Endocrinol..

[49] Lindqvist N., Näpänkangas U., Lindblom J., Hallböök F. (2003). Proopiomelanocortin and Melanocortin Receptors in the Adult Rat Retino-Tectal System and Their Regulation after Optic Nerve Transection. Eur. J. Pharmacol..

[50] Cai S., Yang Q., Hou M., Han Q., Zhang H., Wang J., Qi C., Bo Q., Ru Y., Yang W. (2018). A-Melanocyte-Stimulating Hormone Protects Early Diabetic Retina from Blood-Retinal Barrier Breakdown and Vascular Leakage via MC4R. Cell Physiol. Biochem..

[51] Lee D.J., Taylor A.W. (2013). Both MC5r and A2Ar Are Required for Protective Regulatory Immunity in the Spleen of Post–Experimental Autoimmune Uveitis in Mice. J. Immunol..

[52] Rossi S., Maisto R., Gesualdo C., Trotta M.C., Ferraraccio F., Kaneva M.K., Getting S.J., Surace E., Testa F., Simonelli F. (2016). Activation of Melanocortin Receptors MC1 and MC5 Attenuates Retinal Damage in Experimental Diabetic Retinopathy. Mediat. Inflamm..

[53] Maisto R., Gesualdo C., Trotta M.C., Grieco P., Testa F., Simonelli F., Barcia J.M., D’Amico M., Di Filippo C., Rossi S. (2017). Melanocortin Receptor Agonists MCR1-5 Protect Photoreceptors from High-Glucose Damage and Restore Antioxidant Enzymes in Primary Retinal Cell Culture. J. Cell Mol. Med..

[54] Edwards P.M., Van der Zee C.E.E.M., Verhaagen J., Schotman P., Jennekens F.G.I., Gispen W.H. (1984). Evidence That the Neurotrophic Actions of α-MSH May Derive from Its Ability to Mimick the Actions of a Peptide Formed in Degenerating Nerve Stumps. J. Neurol. Sci..

[55] van de Meent H., Hamers F.P.T., Lankhorst A.J., Joosten E.A.J., Gispen W.H. (1997). Beneficial Effects of the Melanocortin α-Melanocyte-Stimulating Hormone on Clinical and Neurophysiological Recovery after Experimental Spinal Cord Injury. Neurosurgery.

[56] Böhm M., Wolff I., Scholzen T.E., Robinson S.J., Healy E., Luger T.A., Schwarz T., Schwarz A. (2005). α-Melanocyte-Stimulating Hormone Protects from Ultraviolet Radiation-Induced Apoptosis and DNA Damage. J. Biol. Chem..

[57] Kadekaro A.L., Kavanagh R., Kanto H., Terzieva S., Hauser J., Kobayashi N., Schwemberger S., Cornelius J., Babcock G., Shertzer H.G. (2005). α-Melanocortin and Endothelin-1 Activate Antiapoptotic Pathways and Reduce DNA Damage in Human Melanocytes. Cancer Res..

[58] Hill R.P., Wheeler P., MacNeil S., Haycock J.W. (2005). α-Melanocyte Stimulating Hormone Cytoprotective Biology in Human Dermal Fibroblast Cells. Peptides.

[59] Oktar B.K., Yüksel M., Alican I. (2004). The Role of Cyclooxygenase Inhibition in the Effect of α-Melanocyte-Stimulating Hormone on Reactive Oxygen Species Production by Rat Peritoneal Neutrophils. Prostaglandins Leukot. Essent. Fat. Acids.

[60] Delgado R., Carlin A., Airaghi L., Demitri M.T., Meda L., Galimberti D., Baron P., Lipton J.M., Catania A. (1998). Melanocortin Peptides Inhibit Production of Proinflammatory Cytokines and Nitric Oxide by Activated Microglia. J. Leukoc. Biol..

[61] Galimberti D., Baron P., Meda L., Prat E., Scarpini E., Delgado R., Catania A., Lipton J.M., Scarlato G. (1999). α-MSH Peptides Inhibit Production of Nitric Oxide and Tumor Necrosis Factor-α by Microglial Cells Activated with β-Amyloid and Interferon γ. Biochem. Biophys. Res. Commun..

[62] Star R.A., Rajora N., Huang J., Stock R.C., Catania A., Lipton J.M. (1995). Evidence of Autocrine Modulation of Macrophage Nitric Oxide Synthase by α-Melanocyte-Stimulating Hormone. Proc. Natl. Acad. Sci. USA.

[63] Tsatmali M., Graham A., Szatkowski D., Ancans J., Manning P., McNeil C.J., Graham A.M., Thody A.J. (2000). α-Melanocyte-Stimulating Hormone Modulates Nitric Oxide Production in Melanocytes. J. Investig. Dermatol..

[64] Gupta A.K., Diaz R.A., Higham S., Kone B.C. (2000). α-MSH Inhibits Induction of C/EBPβ-DNA Binding Activity and NOS2 Gene Transcription in Macrophages. Kidney Int..

[65] Caruso C., Durand D., Schiöth H.B., Rey R., Seilicovich A., Lasaga M. (2007). Activation of Melanocortin 4 Receptors Reduces the Inflammatory Response and Prevents Apoptosis Induced by Lipopolysaccharide and Interferon-γ in Astrocytes. Endocrinology.

[66] Jung E.J., Han D.J., Chang S.H., Lim D.G., Wee Y.M., Kim J.H., Kim Y.H., Koo S.K., Choi M., Kim S.C. (2007). Protective Effect of Alpha-Melanocyte-Stimulating Hormone on Pancreas Islet Cell Against Peripheral Blood Mononuclear Cell-Mediated Cytotoxicity In Vitro. Transplant. Proc..

[67] Lužnik Z., Sun Z., Nakagawa H., Taylor A.W., Jurkunas U.V., Yin J., Dana R. (2020). Association of α-Melanocyte-Stimulating Hormone with Corneal Endothelial Cell Survival during Oxidative Stress and Inflammation-Induced Cell Loss in Donor Tissue. JAMA Ophthalmol..

[68] Kahale F., Deshpande N., Alemi H., Naderi A., Wang S., Blanco T., Dohlman T.H., Yin J., Jurkunas U.V., Dana R. (2023). Treatment with Neuropeptide Alpha-Melanocyte Stimulating Hormone Suppresses Progression of Fuchs Dystrophy in a UV-Induced Mouse Model. Investig. Ophthalmol. Vis. Sci..

[69] Chu C., Huang Y., Ru Y., Lu X., Zeng X., Liu K., Gan L., Zhang Y., Zhao S. (2021). α-MSH Ameliorates Corneal Surface Dysfunction in Scopolamine-Induced Dry Eye Rats and Human Corneal Epithelial Cells via Enhancing EGFR Expression. Exp. Eye Res..

[70] Chai B., Li J.Y., Zhang W., Newman E., Ammori J., Mulholland M.W. (2006). Melanocortin-4 Receptor-Mediated Inhibition of Apoptosis in Immortalized Hypothalamic Neurons via Mitogen-Activated Protein Kinase. Peptides.

[71] Lee S.Y., Jo S.K., Cho W.Y., Kim H.K., Won N.H. (2004). The Effect of α-Melanocyte-Stimulating Hormone on Renal Tubular Cell Apoptosis and Tubulointerstitial Fibrosis in Cyclosporine A Nephrotoxicity. Transplantation.

[72] Deng J., Hu X., Yuen P.S.T., Star R.A. (2004). α-Melanocyte-Stimulating Hormone Inhibits Lung Injury after Renal Ischemia/Reperfusion. Am. J. Respir. Crit. Care Med..

[73] Taylor A.W., Wayne Streilein J., Cousins S.W. (1994). Alpha-Melanocyte-Stimulating Hormone Suppresses Antigen-Stimulated T Cell Production of Gamma-Lnterferon. Neuroimmunomodulation.

[74] Böhm M., Schulte U., Kalden H., Luger T.A. (1999). Alpha-Melanocyte-Stimulating Hormone Modulates Activation of NF-ΚB and AP-1 and Secretion of Interleukin-8 in Human Dermal Fibroblasts. Ann. N. Y. Acad. Sci..

[75] Böhm M., Schiller M., Ständer S., Seltmann H., Li Z., Brzoska T., Metze D., Schiöth H.B., Skottner A., Seiffert K. (2002). Evidence for Expression of Melanocortin-1 Receptor in Human Sebocytes in Vitro and in Situ. J. Investig. Dermatol..

[76] Manna S.K., Sarkar A., Sreenivasan Y. (2006). α-Melanocyte-Stimulating Hormone down-Regulates CXC Receptors through Activation of Neutrophil Elastase. Eur. J. Immunol..

[77] Morandini R., Boeynaems J.M., Hedley S.J., Macneil S., Ghanem C. (1998). Modulation of ICAM-1 Expression by α-MSH in Human Melanoma Cells and Melanocytes. J. Cell Physiol..

[78] Scholzen T.E., Sunderkötter C., Kalden D.H., Brzoska T., Fastrich M., Fisbeck T., Armstrong C.A., Ansel J.C., Luger T.A. (2003). A-Melanocyte Stimulating Hormone Prevents Lipopolysaccharide-Induced Vasculitis By Down-Regulating Endothelial Cell Adhesion Molecule Expression. Endocrinology.

[79] Böhm M., Eickelmann M., Li Z., Schneider S.W., Oji V., Diederichs S., Barsh G.S., Vogt A., Stieler K., Blume-Peytavi U. (2005). Detection of Functionally Active Melanocortin Receptors and Evidence for an Immunoregulatory Activity of α-Melanocyte-Stimulating Hormone in Human Dermal Papilla Cells. Endocrinology.

[80] Hill R.P., MacNeil S., Haycock J.W. (2006). Melanocyte Stimulating Hormone Peptides Inhibit TNF-α Signaling in Human Dermal Fibroblast Cells. Peptides.

[81] Sarkar A., Sreenivasan Y., Manna S.K. (2003). α-Melanocyte-Stimulating Hormone Induces Cell Death in Mast Cells: Involvement of NF-ΚB. FEBS Lett..

[82] Bhardwaj R., Becher E., Mahnke K., Hartmeyer M., Schwarz T., Scholzen T., Luger T.A. (1997). Evidence for the Differential Expression of the Functional Alpha-Melanocyte-Stimulating Hormone Receptor MC-1 on Human Monocytes. J. Immunol..

[83] Shen L., Barabino S., Taylor A.W., Dana M.R. (2007). Effect of the Ocular Microenvironment in Regulating Corneal Dendritic Cell Maturation. Arch. Ophthalmol..

[84] Gonindard C., Goigoux C., Hollande E., D’Hinterland L.D. (1996). The Administration of an α-MSH Analogue Reduces the Serum Release of IL-1α and TNFα Induced by the Injection of a Sublethal Dose of Lipopolysaccharides in the BALB/c Mouse. Pigment Cell Res..

[85] Han D., Tian Y., Zhang M., Zhou Z., Lu J. (2007). Prevention and Treatment of Experimental Autoimmune Encephalomyelitis with Recombinant Adeno-Associated Virus-Mediated α-Melanocyte-Stimulating Hormone-Transduced PLP139-151-Specific T Cells. Gene Ther..

[86] Oktar B.K., Ercan F., Yegen B.Ç., Alican I. (2000). The Effect of α-Melanocyte Stimulating Hormone on Colonic Inflammation in the Rat. Peptides.

[87] Bhardwaj R.S., Schwarz A., Becher E., Mahnke K., Aragane Y., Schwarz T., Luger T.A. (1996). Pro-Opiomelanocortin-Derived Peptides Induce IL-10 Production in Human Monocytes. J. Immunol..

[88] Redondo P., García-Foncillas J., Okroujnov I., Bandrés E. (1998). α-MSH Regulates Interleukin-10 Expression by Human Keratinocytes. Arch. Dermatol. Res..

[89] Najafi S., Elbasiony E., Zidan A.A., Yin J. (2023). Alpha-Melanocyte-Stimulating Hormone (α-MSH) Suppresses Corneal Angiogenesis and Inflammation. Investig. Ophthalmol. Vis. Sci..

[90] Rheins L.A., Cotleur A.L., Kleier R.S., Hoppenjans W.B., Saunder D.N., Nordlund J.J. (1989). Alpha-Melanocyte Stimulating Hormone Modulates Contact Hypersensitivity Responsiveness in C57/BL6 Mice. J. Investig. Dermatol..

[91] Grabbe S., Bhardwaj R.S., Mahnke K., Simon M.M., Schwarz T., Luger T.A. (1996). Alpha-Melanocyte-Stimulating Hormone Induces Hapten-Specific Tolerance in Mice. J. Immunol..

[92] Hamrah P., Haskova Z., Taylor A.W., Zhang Q., Ksander B.R., Dana M.R. (2009). Local Treatment with Alpha-Melanocyte Stimulating Hormone Reduces Corneal Allorejection. Transplantation.

[93] Tahvildari M., Inomata T., Amouzegar A., Dana R. (2018). Regulatory T Cell Modulation of Cytokine and Cellular Networks in Corneal Graft Rejection. Curr. Ophthalmol. Rep..

[94] Taylor A.W., Dinwoodie I.R., Linderman S.E. (2014). Retinal Pigment Epithelium Activation of Alternative Macrophages. Investig. Ophthalmol. Vis. Sci..

[95] Haycock J.W., Wagner M., Morandini R., Ghanem G., Rennie I.G., Mac Neil S. (1999). α-Melanocyte-Stimulating Hormone Inhibits NF-ΚB Activation in Human Melanocytes and Melanoma Cells. J. Investig. Dermatol..

[96] Moustafa M., Szabo M., Ghanem G.E., Morandini R., Kemp E.H., MacNeil S., Haycock J.W. (2002). Inhibition of Tumor Necrosis Factor-α Stimulated NFκB/P65 in Human Keratinocytes by α-Melanocyte Stimulating Hormone and Adrenocorticotropic Hormone Peptides. J. Investig. Dermatol..

[97] Teare K.A., Pearson R.G., Shakesheff K.M., Haycock J.W. (2004). α-MSH Inhibits Inflammatory Signalling in Schwann Cells. Neuroreport.

[98] Ichiyama T., Zhao H., Catania A., Furukawa S., Lipton J.M. (1999). α-Melanocyte-Stimulating Hormone Inhibits NF-ΚB Activation and IκBα Degradation in Human Glioma Cells and in Experimental Brain Inflammation. Exp. Neurol..

[99] Zou L., Sato N., Kone B.C. (2004). Amelanocyte Stimulating Hormone Protects against H_2_O 2-Induced Inhibition of Wound Restitution in IEC-6 Cells via a Syk Kinase- and NF-Κβ-Dependent Mechanism. Shock.

[100] Puri S., Kenyon B.M., Hamrah P. (2022). Immunomodulatory Role of Neuropeptides in the Cornea. Biomedicines.

[101] Shi M., Zhang L., Ye E.A., Wang A., Li G., Chen L. (2020). Aqueous humor induces lymphatic regression on the ocular surface. Ocul. Surf..

[102] Cheng P.W., Tsai P.J., Tai M.H., Bee Y.S. (2021). Therapeutic Effect of α-Msh in Primary Cultured Orbital Fibroblasts Obtained from Patients with Thyroid Eye Disease. Int. J. Mol. Sci..

[103] Yin J., Liu L., Pang K. (2021). Corneal Nerves Modulate Angiogenesis via Secreted Neuropeptides. Investig. Ophthalmol. Vis. Sci..

[104] Yin J., Liu L., Pang K. (2022). Peripheral Sensory Nerves Inhibit Corneal Angiogenesis via Alpha-Melanocyte-Stimulating Hormone. Investig. Ophthalmol. Vis. Sci..

[105] Gipson I.K. (2007). The Ocular Surface: The Challenge to Enable and Protect Vision. The Friedenwald Lecture. Investigative Ophthalmology and Visual Science.

[106] Müller L.J., Marfurt C.F., Kruse F., Tervo T.M.T. (2003). Corneal Nerves: Structure, Contents and Function. Exp. Eye Res..

[107] Van Der Kraan M., Adan R.A.H., Entwistle M.L., Gispen W.H., Burbach J.P.H., Tatro J.B. (1998). Expression of Melanocortin-5 Receptor in Secretory Epithelia Supports a Functional Role in Exocrine and Endocrine Glands. Endocrinology.

[108] Jahn R., Padel U., Porsch P.H., Soling H.-D. (1982). Adrenocorticotropic Hormone and A-Melanocyte-Stimulating Hormone Induce Secretion and Protein Phosphorylation in the Rat Lacrimal Gland by Activation of a CAMP-Dependent Pathway. Eur. J. Biochem..

[109] Evans D., Kenyon K., Ousler G., Watson M., Vollmer P., McLaurin E.B., Torkildsen G., Winters J., Dodd J., Jordan R. (2023). Efficacy and Safety of the Melanocortin Pan-Agonist PL9643 in a Phase 2 Study of Patients with Dry Eye Disease. J. Ocul. Pharmacol. Ther..

[110] Pavan J., Lukenda A., Štambuk N., Konjevoda P., Kaštelan S., Ćurković M. (2012). Effects of Alpha-MSH on Corneal Epithelial Lesions in Rats. Coll. Antropol..

[111] Naveh N., Marshall J. (2001). Melanocortins Are Comparable to Corticosteroids as Inhibitors of Traumatic Ocular Inflammation in Rabbits. Graefe’s Arch. Clin. Exp. Ophthalmol..

[112] Zhang Z., Ma J., Yao K., Yin J. (2012). Alpha-Melanocyte Stimulating Hormone Suppresses the Proliferation of Human Tenon’s Capsule Fibroblast Proliferation Induced by Transforming Growth Factor Beta 1. Mol. Biol..

[113] Kleiner S., Braunstahl G.J., Rüdrich U., Gehring M., Eiz-Vesper B., Luger T.A., Steelant B., Seys S.F., Kapp A., Böhm M. (2016). Regulation of Melanocortin 1 Receptor in Allergic Rhinitis in Vitro and in Vivo. Clin. Exp. Allergy.

[114] Webering S., Lunding L.P., Vock C., Schröder A., Gaede K.I., Herzmann C., Fehrenbach H., Wegmann M. (2019). The Alpha-Melanocyte-Stimulating Hormone Acts as a Local Immune Homeostasis Factor in Experimental Allergic Asthma. Clin. Exp. Allergy.

[115] Lee D.J., Biros D.J., Taylor A.W. (2009). Injection of an Alpha-Melanocyte Stimulating Hormone Expression Plasmid Is Effective in Suppressing Experimental Autoimmune Uveitis. Int. Immunopharmacol..

[116] Nishida T., Miyata S., Itoh Y., Mizuki N., Ohgami K., Shiratori K., Ilieva I.B., Ohno S., Taylor A.W. (2004). Anti-Inflammatory Effects of Alpha-Melanocyte-Stimulating Hormone against Rat Endotoxin-Induced Uveitis and the Time Course of Inflammatory Agents in Aqueous Humor. Int. Immunopharmacol..

[117] Shiratori K., Ohgami K., Ilieva I.B., Koyama Y., Yoshida K., Ohno S. (2004). Inhibition of Endotoxin-Induced Uveitis and Potentiation of Cyclooxygenase-2 Protein Expression by α-Melanocyte-Stimulating Hormone. Investig. Ophthalmol. Vis. Sci..

[118] Ng T.F., Kitaichi N., Taylor A.W. (2007). In Vitro-Generated Autoimmune Regulatory T Cells Enhance Intravitreous Allogeneic Retinal Graft Survival. Investig. Ophthalmol. Vis. Sci..

[119] Teshigawara K., Takahashi S., Boswell T., Li Q., Tanaka S., Takeuchi S. (2001). Identification of Avian α-Melanocyte-Stimulating Hormone in the Eye: Temporal and Spatial Regulation of Expression in the Developing Chicken. J. Endocrinol..

[120] Naveh N. (2003). Melanocortins Applied Intravitreally Delay Retinal Dystrophy in Royal College of Surgeons Rats. Graefe’s Arch. Clin. Exp. Ophthalmol..

